# Steering Angle Assisted Vehicular Navigation Using Portable Devices in GNSS-Denied Environments

**DOI:** 10.3390/s19071618

**Published:** 2019-04-04

**Authors:** Mohamed Moussa, Adel Moussa, Naser El-Sheimy

**Affiliations:** 1 Geomatics Engineering Department, University of Calgary, Calgary, AB T2N 1N4, Canada; amelsaye@ucalgary.ca (A.M.); elsheimy@ucalgary.ca (N.E.-S.); 2 Department of Electrical Engineering, Port Said University, Port Said 42523, Egypt

**Keywords:** Consumer Portable Devices, Land Vehicle Navigation, GNSS/IMU integration, Steering Angle Estimation

## Abstract

Recently, land vehicle navigation, and especially by the use of low-cost sensors, has been the object of a huge level of research interest. Consumer Portable Devices (CPDs) such as tablets and smartphones are being widely used by many consumers all over the world. CPDs contain sensors (accelerometers, gyroscopes, magnetometer, etc.) that can be used for many land vehicle applications such as navigation. This paper presents a novel approach for estimating steering wheel angles using CPD accelerometers by attaching CPDs to the steering wheel. The land vehicle change of heading is then computed from the estimated steering wheel angle. The calculated change of heading is used to update the navigation filter to aid the onboard Inertial Measurement Unit (IMU) through the use of an Extended Kalman Filter (EKF) in GNSS-denied environments. Four main factors that may affect the steering wheel angle accuracy are considered and modeled during steering angle estimations: static onboard IMU leveling, inclination angle of the steering wheel, vehicle acceleration, and vehicle inclination. In addition, these factors are assessed for their effects on the final result. Therefore, three methods are proposed for steering angle estimation: non-compensated, partially-compensated, and fully-compensated methods. A road experimental test was carried out using a Pixhawk (PX4) navigation system, iPad Air, and the OBD-II interface. The average Root Mean Square Error (RMSE) of the change of heading estimated by the proposed method was 0.033 rad/s. A navigation solution was estimated while changes of heading and forward velocity updates were used to aid the IMU during different GNSS signal outages. The estimated navigation solution is enhanced when applying the proposed updates to the navigation filter by 91% and 97% for 60 s and 120 s of GNSS signal outage, respectively, compared to the IMU standalone solution.

## 1. Introduction

Recently, Inertial Measurement Units (IMUs) and Global Navigation Satellite Systems (GNSS) have been widely used to provide accurate and reliable navigation information (i.e., altitude, velocity, and position). GNSS has long-term stability in ideal conditions but has certain limitations in urban areas (e.g., congested areas), inside tunnels and under heavy tree canopies. IMU is completely self-contained and autonomous, but suffers from accuracy degradation over time [1]. The integration of GNSS and IMU can maximize the respective advantages and minimize the individual drawbacks, providing a more consistent navigation solution [2]. Furthermore, many sensors can also be used to aid the IMU, such as Light Detection and Ranging (LiDAR) [3], cameras [4], odometers [5], magnetometers [6], barometers, ultrasonic sensors [7], etc. However, there are some limitations to these aiding sources which will be discussed in the Problem Formulation section.

Consumer portable devices (CPDs) such as tablets and smartphones contain many sensors—such as GNSS receivers, IMUs, barometers and magnetometers—that can be used in navigation [8]. CPDs, therefore, offer all the measurements needed to provide full navigation states. This paper introduces a new way of using CPDs to measure heading changes by estimating the steering angle as an update in the navigation estimation filter. The steering angle information may be determined from the Controller Area Network (CAN) bus, using Steering Angle Sensors (SAS). However, this information is not typically provided by commercial OBD-II units, and it requires additional customized hardware and software designs [9,10,11].

## 2. Related Works

The last decade has seen major research on the use of the consumer portable devices in land vehicle applications such as navigation applications [12,13], road condition analyses [14], traffic states [15], and insurance telematics [16]. Different smartphone applications for land vehicles are summarized in review paper [17].

CPDs have also been used in land vehicle navigation applications. For example in Ref. [18], the MEMS IMU inside an iPhone 4 was integrated with GPS along with Non-Holonomic Constraint (NHC) for car navigation using loosely-coupled integration through the use of an Extended Kalman Filter (EKF). The authors concluded that the accuracy of the attitude angle is in the range of 2 degrees for heading and less than 1.5 degrees for roll and pitch, while the position accuracy reached 30 m after 30 s of GNSS signal outage. Three-dimensional accelerometers and one gyroscope were used by Ref. [19], in addition to a probabilistic map-matching algorithm to tune the navigation solution and calibrate the inertial sensors errors.

Automatic parking positioning (Park Sense) has been proposed by Ref. [20] by sensing in-vehicle magnetic field variations using a smartphone magnetometer. Precise magnetic fingerprinting-based outdoor localization has been proposed by Ref. [21]. Smartphone gyroscopes have been integrated with a vehicle’s CAN bus for speed information to provide a navigation solution during GNSS signal outages using a Kalman Filter [22]. A vehicle navigation system was proposed by Ref. [23] using the smartphone rear camera and IMU to identify the position of a car within the lane using computer vision techniques, to an accuracy of 88.5%. Smartphone sensors (GNSS, and IMU) have been used by [24] for lane localization through machine-learning based on a Support Vector Machine (SVM) -based lane-change identification algorithm to detect lane changes to an accuracy of 98 %. In Ref. [25], a mobile mapping system using a smart phone GNSS, MEMS IMU, an odometer (CAN bus), and Velodyne HDL-32e LiDAR was developed using the SLAM and loop closure method, where the output maps were used for self-driving applications. VeMap, a roadmap construction system based on smartphones inside vehicles, was proposed by Ref. [26]; in this approach, multiple sensors were fused together for navigation-denied GNSS environments such as underground parking.

Most approaches reported in the literature have used gyroscopes as the main sensors for estimating steering wheel angles. However, these methods are very sensitive to gyro errors. Therefore, some research has integrated other sensors to compensate for these errors. Ref. [27] used gyroscopes to estimate steering wheel angles and incorporated accelerometer and magnetometer measurements to reduce biases and errors due to the gyroscope errors through the use of an Extended Kalman Filter; the main problem here is introducing more sensors, as well as minimizing errors and biases, to the system when estimating the steering wheel angle. Furthermore, this work [27] didn’t compensate for vehicle inclination, which may affect the accuracy of the estimated steering wheel angle. Other related works have used gyroscope data from smartwatches to measure wrist rotation; in this way, the steering wheel angle can be estimated in unsafe driving detection applications [28]. In Ref. [29], both the accelerometer and gyroscope measurements have been integrated through a weighted average method to estimate steering wheel angles where the gyroscope was the main sensor. However, the vehicle inclination and acceleration, static leveling, and the steering wheel inclination angles are not considered for the computation of steering wheel angles.

Other researchers have used the accelerometers as the main sensors to estimate steering wheel angles. Ref. [30] used accelerometers only to estimate the steering wheel angle. The steering wheel inclination angle is considered here, but vehicle inclination is not taken into consideration during estimations. Moreover, the acceleration and the deceleration of the vehicle (the non-gravity-related acceleration) were not modeled in the solution. A low-cost driving data acquisition system for different land vehicles, BigRoad, was proposed by [11]. These data, comprising steering wheel angle, speed, and conditions of the surrounding environment of the vehicle, are obtained using smartphones and IMUs installed inside the vehicle. However, the method of estimating the steering angle using Invensense MPU-9150 accelerometers takes into account neither the leveling of the device in a static state nor the vehicle inclination. Moreover, the steering wheel inclination was estimated using gyroscopes which require rotation maneuvers. Finally, they didn’t employ the estimated steering wheel angle in navigation states estimations.

## 3. Problem Formulation and Objective

Many aiding sensors have been discussed in previous papers, as described in the introduction. However, most of these have some limitations; for example, LiDAR aiding is expensive and typically needs high storage and high processing, and may be affected by weather conditions. On the other hand, vision-aided navigation systems suffer from weather and lighting conditions in addition to scale issues when using a single camera. Magnetometers may be used as aiding sensors for IMUs, but they suffer from many issues such as the magnetic interference (soft and hard iron effects) [31], biases and scale factors, in addition to environmental magnetic disturbances that greatly affect the magnetometers [32], especially in land vehicle navigation applications.

As the accelerometer measures only the linear acceleration of the device, it could be used to estimate both the roll and pitch angles, but it cannot observe the heading (azimuth) or its change. Gyroscopes are the most commonly-used sensor used for heading/azimuth change measurements, but such measurements are typically contaminated by a high level of errors (bias + scale factor, noise) on account of the consumer-level sensors used in CPDs.

The main objective of this paper is to introduce a new way to use CPD sensors to update IMUs in land vehicle navigation estimation filters. The idea is based on estimating the steering wheel angle using CPD accelerometers mounted on the steering wheel. This steering angle is converted to change of heading information for the vehicle motion, which can be considered as a heading update to the navigation solution through EKF to limit IMU drift during GNSS signal outages.

## 4. Methodology

**Figure 1:**
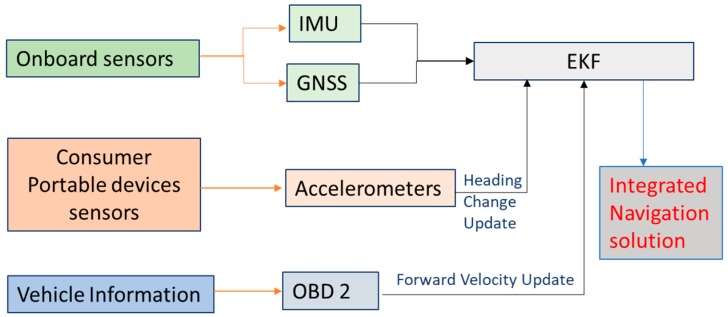
Consumer Portable devices/on-board navigation sensor integration architecture.

This paper introduces a new approach for estimating the navigation states of land vehicles in GNSS-denied environment by integrating CPD sensors with onboard sensors along with vehicle information through EKF. The navigation system architecture is shown in Figure 1.

The system will obtain information from three sources:

(1) An integrated GNSS and IMU to estimate the full vehicle navigation states. In this integration, the GNSS provides the absolute position and velocity. This integration is called the onboard sensors.
(2) Vehicle information which can be obtained via the On-Board Diagnostics (OBD-II). The OBD-II provides forward velocity of the vehicle direction which can be used as a velocity update to limit the IMU velocity and position drift during GNSS signal outages.
(3) A new idea for using CPD sensors to estimate the heading of the vehicle. The main idea depends on mounting the portable device onto the steering wheel of the vehicle to estimate the rotation angle of the steering wheel (called steering angle), using the self-contained tri-axial accelerometers of the CPD. This steering angle can then be used to compute changes of heading of land vehicles.

It is very well known that heading errors of IMU constitute one of the major sources of position drifts. For example, gyroscope bias introduces heading errors through the following equations:

(1) $$\delta A=\frac{b_{\omega }}{\omega_{e}\cos\phi }$$

(2) $$\delta P=\left(\frac{1}{6}\right)b_{\omega }gt^{3}$$

where *δA* is the heading error, *b_w_* is the gyroscope bias, *ω_e_* is the Earth’s rotation (15 °/hr), and *φ* is the latitude of the vehicle. *δP* is the position error due to the gyroscope drift (*b_ω_*), *g* is the gravity acceleration, *t* is the time since the IMU work in stand-alone mode. For example, for a gyroscope with a drift of 0.1 °/s, after 10 s, the position error will reach around 2.85 m. Therefore, constraining the heading drift will dramatically reduce position errors during GNSS signal outages. However, there are four main factors that should be compensated for in order to better estimate the steering angle, i.e.,:

(1) The static leveling of the GNSS/IMU integrated system.
(2) The inclination angle of the steering wheel.
(3) The acceleration of the vehicle.
(4) The vehicle inclination.

These factors are discussed in detail in the next subsections.

### 4.1. Different Used Coordinate Frames

**Figure 2:**
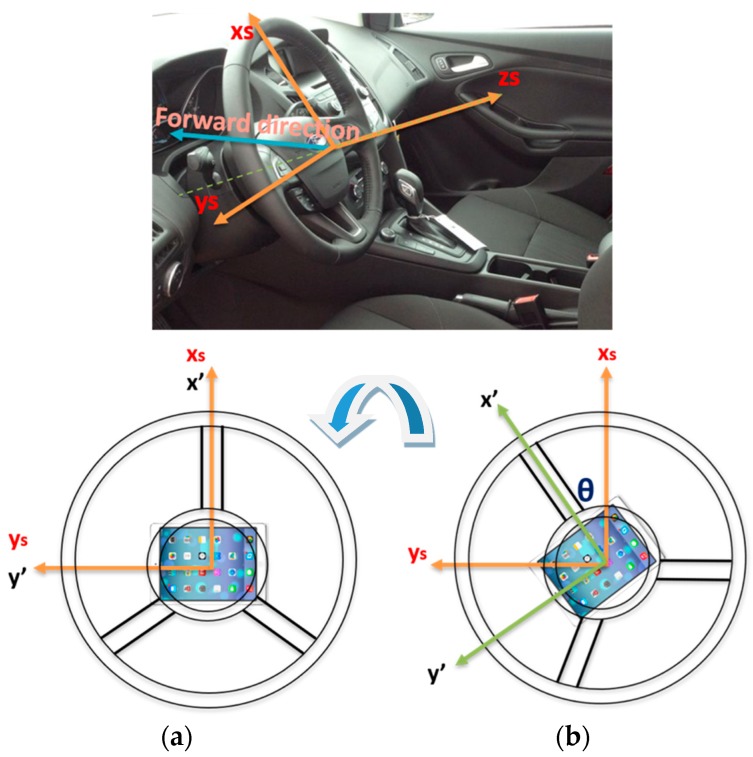
Steering fixed frame and the CPD frame. (**a**) The steering fixed and the CPD frames coincide for a 0° steering angle; (**b**) the steering angle and the CPD frames for steering angle (*θ*).

**Figure 3:**
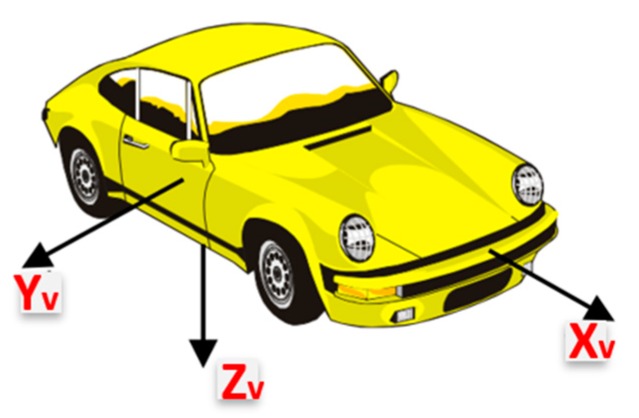
Vehicle frame (Body frame).

In this research, four main coordinate frames are used:

1. The steering wheel frame, which is a fixed frame, is defined at a 0° steering angle, where the *x*-axis is in the up direction and is defined as *x_s_*, while *y_s_* is perpendicular to *x_s_* and in the same plane of the steering wheel and pointing to the left direction, while *z_s_* is perpendicular to the steering wheel plane (*x_s_*-*y_s_* plane).
2. The CPD frame comprises the 3-axis accelerometers frame which rotates with the steering wheel where *x*’ and *y*’ are in the upper and left direction respectively while *z*’ is perpendicular to the CPD plane defined by *x*’ and *y*’. When the steering angle equals zero, both frames coincide with each other. Figure 2 shows the relationship between both frames at two different steering angles: zero and (*θ*), respectively.
3. Body frame (vehicle frame). The IMU body frame is defined by the axis of the accelerometers of the IMU, and in this research, it is aligned with the vehicle frame in which the vehicle forward direction is aligned with the *x*-axis of the onboard IMU (*X_v_*), while the *y*-axis (*Y_v_*) of the onboard IMU coincides with the vehicle lateral direction, and finally, the IMU *z*-axis (*Z_v_*) is directed downwards, as shown in Figure 3.
4. The navigation frame is defined as the local level frame (North, East, and Down directions) in which the orientation and the velocity are estimated.

### 4.2. Static Leveling of Vehicle Onboard IMU

The inclination of the vehicle in a stationary condition should be taken into consideration before estimating the steering angle. This inclination is caused by the presence of the vehicle on an up- or down-hill road. The vehicle pitch and roll angles are functions of the earth’s gravity acceleration (*g*) components sensed by the *x*, and *y* onboard IMU accelerometers (forward and lateral direction of the vehicle), as given in Equations (3) and (4).

(3) $$r=\sin^{-1}(\frac{a_{Y_{v}}}{g})$$

(4) $$p=\sin^{-1}(\frac{a_{X_{v}}}{g})$$

Generally, the pitch angle is much more important than the roll angle in regular land vehicle navigation because cars may experience large changes in elevation on normal roads (e.g., on mountainous roads). Therefore, cars’ motion can experience significant pitch angles. On the other hand, the roll can be neglected because of its very small values under normal road conditions.

### 4.3. Inclination Angle of Steering Wheel

**Figure 4:**
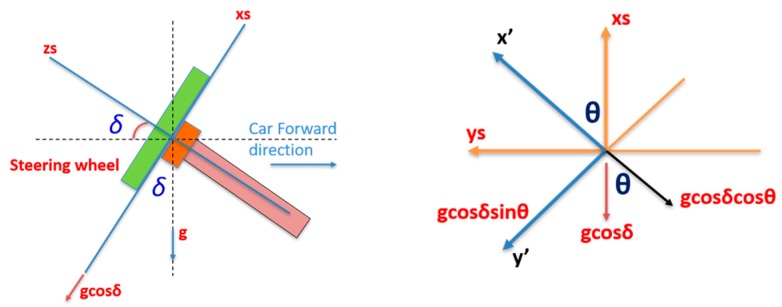
Steering wheel inclination angle.

The second factor which should be compensated for when determining the steering angle is the inclination angle of the steering wheel (*δ*), as shown in Figure 4. The steering wheel inclination angle is the angle between the *z_s_* axis and the car forward direction axis (*X_v_*).

There are two methods that can be used to estimate the steering wheel inclination angle; the first depends on the *x* and *y* accelerometers of the CPD that lie on the same plane as the steering wheel. The following equations show the relationship between the *x* and *y* accelerometers with gravity acceleration, the steering wheel inclination angle (*δ*) and the steering angle (*θ*).

(5) $$\begin{array}{l} a_{x^{\prime }}=g\cos\delta \cos\theta \\ a_{y^{\prime }}=g\cos\delta \sin\theta \end{array}$$

(6) $$\begin{array}{l} {a_{x^{\prime }}}^{2}=g^{2}\cos^{2}\delta \cos^{2}\theta \\ {a_{y^{\prime }}}^{2}=g^{2}\cos^{2}\delta \sin^{2}\theta \end{array}$$

(7) $${a_{x^{\prime }}}^{2}+{a_{y^{\prime }}}^{2}=g^{2}\cos^{2}\delta [\cos^{2}\theta +\sin^{2}\theta ]$$

The inclination angle can be estimated from Equation (8).

(8) $$\delta =\cos^{-1}(\sqrt{\frac{a_{x^{\prime }}^{2}+a_{y^{\prime }}^{2}}{g^{2}}})$$

The second method uses the third accelerometer that is perpendicular to the steering wheel plane (i.e., the z accelerometer), as shown in Equations (9) and (10).

(9) $$a_{z^{\prime }}=g\sin\delta$$

(10) $$\delta =\sin^{-1}(\frac{a_{z^{\prime }}}{g})$$

**Figure 5:**
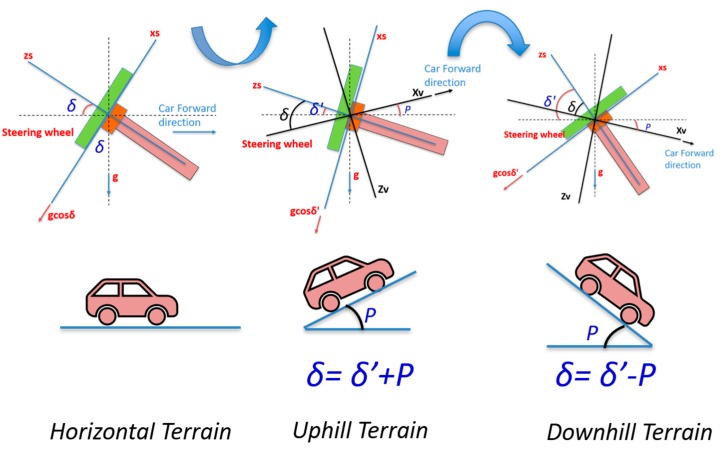
Different terrain types of land vehicle starting.

However, in Equations (9) and (10), the vehicle is assumed to be on horizontal terrain when calculating the steering wheel inclination (i.e., the body or the vehicle frame coincides with the direction of gravity and the horizontal axis). Figure 5 shows the different cases for calculating the steering wheel inclination (*δ*) based on the pitch (*P*) of the vehicle at the start of the navigation.

For uphill or downhill roads, the non-compensated steering wheel inclination angle (*δ*’) is calculated, instead of the steering wheel inclination angle (*δ*), using Equations (8) and (10) (*δ*’ is the angle between the *z_s_* axis and the horizontal plane). The steering wheel inclination angle should be compensated for by the pitch angle which was determined during the static leveling process, as given in Equations (11) and (12) for the two cases of the uphill and downhill terrains respectively.

(11) $$\delta =\delta^{\prime }+P$$

(12) $$\delta =\delta^{\prime }-P$$

Finally, the estimated inclination angle from the two previous methods (Equations (8) and (10)) are fused into a single estimate to make use of all used accelerometer sensors through least squares adjustment.

### 4.4. Steering Angle Estimation Using Portable Devices Accelerometers

**Figure 6:**
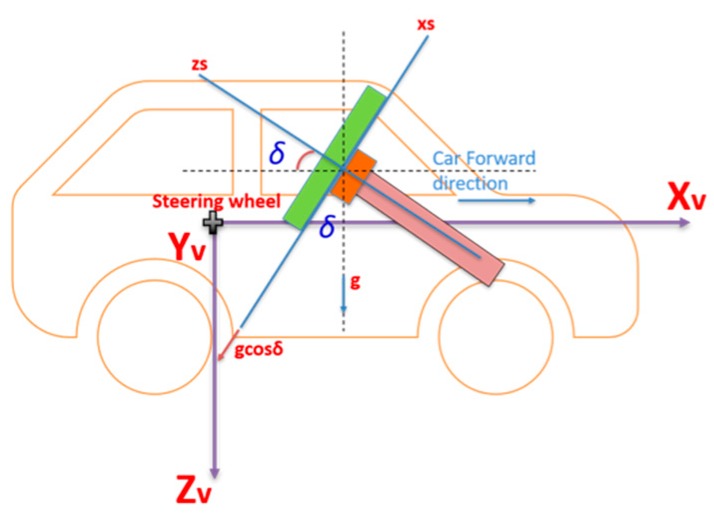
Steering angle estimation using CPD accelerometers.

The relationship between the vehicle’s frame and the fixed steering frame can be derived as shown in Figure 6.

The acceleration in the *x_s_* direction senses three components, i.e., the forward and vertical vehicle acceleration and the gravity acceleration, after compensating for all of them with the inclination angle. On the other hand, the acceleration in direction *y_s_* measures the lateral vehicle acceleration only. Meanwhile, the measured acceleration in the *z_s_* direction includes components of the forward and the vertical vehicle acceleration, as shown in Equations (13)–(15).

(13) $$a_{x_{s}}=a_{X_{v}}\sin\delta -g\cos\delta -(a_{Z_{v}}+g)\cos\delta$$

(14) $$a_{y_{s}}=-a_{Y_{v}}$$

(15) $$a_{z_{s}}=-a_{X_{v}}\cos\delta -(a_{Z_{v}}+g)\sin\delta$$

The relationship between the fixed and the rotating steering frames is described in Equation (16).

(16) $$\left[\begin{array}{c} a_{x^{\prime }}\\ a_{y^{\prime }} \end{array}\right]=\left[\begin{array}{cc} \cos\theta & \sin\theta \\ -\sin\theta & \cos\theta \end{array}\right]\left[\begin{array}{c} a_{x_{s}}\\ a_{y_{s}} \end{array}\right]$$

The relation between the vehicle frame and the rotating steering frame is described in Equations (17) and (18).

(17) $$a_{x^{\prime }}=\cos\theta [a_{X_{v}}\sin\delta -g\cos\delta -(a_{Z_{v}}+g)\cos\delta ]-a_{Y_{v}}\sin\theta$$

(18) $$a_{y^{\prime }}=-a_{Y_{v}}\cos\theta -\sin\theta [a_{X_{v}}\sin\delta -g\cos\delta -(a_{Z_{v}}+g)\cos\delta ]$$

The steering angle can be derived using Equations (19) to (26).

(19) $$a_{x^{\prime }}=a_{x_{s}}\cos\theta +a_{y_{s}}\sin\theta$$

(20) $$a_{y^{\prime }}=-a_{xs}\sin\theta +a_{ys}\cos\theta$$

Dividing Equation (20) by Equation (19) gives:

(21) $$\frac{a_{y^{\prime }}}{a_{x^{\prime }}}=\frac{-a_{x_{s}}\sin\theta +a_{y_{s}}\cos\theta }{a_{x_{s}}\cos\theta +a_{y_{s}}\sin\theta }$$

(22) $$\frac{a_{y^{\prime }}}{a_{x^{\prime }}}=\frac{-a_{x_{s}}\tan\theta +a_{y_{s}}}{a_{x_{s}}+a_{y_{s}}\tan\theta }$$

Cross multiplication between the left and right-hand sides of Equation (22).

(23) $$a_{x^{\prime }}[-a_{x_{s}}\tan\theta +a_{y_{s}}]=a_{y^{\prime }}[a_{x_{s}}+a_{y_{s}}\tan\theta ]$$

(24) $$-a_{x^{\prime }}a_{x_{s}}\tan\theta +a_{x^{\prime }}a_{y_{s}}-a_{y^{\prime }}a_{x_{s}}-a_{y^{\prime }}a_{y_{s}}\tan\theta =0$$

(25) $$a_{x^{\prime }}a_{x_{s}}\tan\theta +a_{y^{\prime }}a_{y_{s}}\tan\theta =a_{x^{\prime }}a_{y_{s}}-a_{y^{\prime }}a_{x_{s}}$$

(26) $$\tan\theta =\frac{a_{x^{\prime }}a_{y_{s}}-a_{y^{\prime }}a_{x_{s}}}{a_{x^{\prime }}a_{x_{s}}+a_{y^{\prime }}a_{y_{s}}}$$

Finally, Equation (27) leads to the steering angle.

(27) $$\tan\theta =\frac{-a_{x^{\prime }}a_{Y_{v}}-a_{y^{\prime }}[a_{X_{v}}\sin\delta -g\cos\delta -(a_{Z_{v}}+g)\cos\delta ]}{a_{x^{\prime }}[a_{X_{v}}\sin\delta -g\cos\delta -(a_{Z_{v}}+g)\cos\delta ]-a_{y^{\prime }}a_{Y_{v}}}$$

### 4.5. Vehicle Inclination

When the car moves up- or down-hill, the *x* accelerometer of the onboard IMU senses a partial component of the gravity acceleration vector while the z accelerometer senses the rest of the vector. Equations (28)–(30) show this effect.

(28) $$a_{X_{v}^{\prime }}=a_{X_{v}}-g\sin p$$

(29) $$a_{Y_{v}^{\prime }}=a_{Y_{v}}$$

(30) $$a_{Z_{v}^{\prime }}=a_{Z_{v}}+g\cos p$$

where the pitch angle is estimated using GNSS/IMU integration solution of the onboard sensors. Such a compensation accounts for the dynamic change of the vehicle inclination.

Therefore, the general formula for determining the steering angle is expressed as follows in Equation (31).

(31) $$\tan\theta =\frac{-a_{x^{\prime }}a_{Y_{v}^{\prime }}-a_{y^{\prime }}[a_{X_{v}^{\prime }}\sin\delta -g\cos\delta -a_{Z_{v}^{\prime }}\cos\delta ]}{a_{x^{\prime }}[a_{X_{v}^{\prime }}\sin\delta -g\cos\delta -a_{Z_{v}^{\prime }}\cos\delta ]-a_{y^{\prime }}a_{Y_{v}^{\prime }}}$$

This general equation takes into consideration all the factors that may affect the steering angle estimation, i.e., the static leveling of the onboard IMU, the inclination angle of the steering wheel, the land vehicle acceleration, and the dynamic change of the vehicle’s inclination.

**Figure 7:**
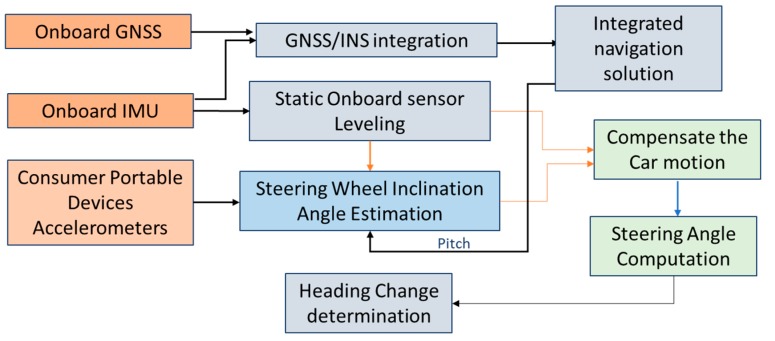
Fully-Compensated steering angle and heading change estimation.

Figure 7 shows a flow chart of the full compensation for the steering angle estimation, in which all the four aforementioned factors are implemented to estimate the steering angle.

### 4.6. Change of Heading Computaion

Changes of heading of the vehicle motion are determined after estimating the steering angle by multiplying this angle by the Vehicle Steering Ratio (VSR). The VSR is defined as the ratio between the steering wheel angle and the wheel (tire) angle (which is the heading change of the vehicle). This ratio varies from one car to another, depending on the type and model of the car; it ranges between (1:12) to (1:24) [33]. This ratio is constant for a certain type and model of vehicle. Equation (32) shows the relationship between the change of heading (Δ*θ_vehicle_*) and the steering angle (*θ_steering_*) [11].

(32) $$\Delta \theta_{vehicle}=(VSR)\theta_{steering}$$

## 5. Land Vehicle Navigation Estimation

Land vehicle navigation states are usually estimated through GNSS/IMU integration to determine the navigation states which include: 3D position, 3D velocity and the orientation of the vehicle. The next subsection describes the GNSS/IMU integration through EKF, while the second subsection describes the proposed updates to estimate the land vehicle navigation states.

### 5.1. GNSS/IMU Integration

GNSS/IMU integration can be implemented through loosely-, tightly-, or deeply-coupled integration schemes [34]. In this research, a loosely-coupled integration scheme is used where GNSS provides both position and velocity updates to the IMU navigation solution through EKF.

KF is based on two main models: the system model and the observation model. The system model describes changes in states over time [35]. The continuous time system model is expressed in Equation (33).

(33) $$\dot{x}(t)=F(t)x(t)+G(t)w(t)$$

where $\dot{x}$ is the time rate of change of the state vector, *F* is the dynamics matrix, *x* is the state vector, *G* is the shaping matrix, and *w* is white noise. The system may be described in a discrete time model [36], as given in Equation (34).

(34) $$x_{k+1}=\phi_{k,k+1}x_{k}+w_{k}$$

where *ϕ* is the transition matrix which is calculated using the dynamics matrix, as given in Equation (35), where Δ*t* is the discrete time interval and *I* is an identity matrix.

(35) ϕ=(I+FΔt)

The observation model is described in Equation (36), where *z_k_* is the observation vector, *H_k_* is the design matrix and *η* is the measurement noise.

(36) $$z_{k}=H_{k}x_{k}+\eta_{k}$$

KF is divided into two main stages: the prediction stage and the update stage [36]. The system model is responsible for the prediction stage, in which the error states (*x*_k_) and its covariance matrix (*P*_k_) are predicted, as in Equations (37) and (38).

(37) $$x_{k}^{-}=\phi_{k,k-1}{\overset{⌢}{x}}_{k-1}^{+}$$

(38) $$P_{k}^{-}=\phi_{k,k-1}P_{k-1}^{+}\phi_{k,k-1}^{T}+Q_{k-1}$$

where (−) define the predicted values and (+) define the updated values, (*Q_k_*_−1_) is the process noise matrix which describes the degree of confidence in the system model.

The second stage is the update stage, in which the Kalman gain (*K_k_*) is computed as in Equation (39), which is a function of the degree of confidence of the observation model (*R_k_*), as well as the degree of confidence of the system model, which is represented in the predicted covariance matrix of the error states. Next, the updated error states, as well as the updated covariance matrix, are computed [36], as shown in Equations (40) and (41).

(39) $$K_{k}=P_{k}^{-}H_{k}^{T}{\left[H_{k}P_{k}^{-}H_{k}^{T}+R_{k}\right]}^{-1}$$

(40) $${\overset{⌢}{x}}_{k}^{+}={\overset{⌢}{x}}_{k}^{-}+K_{k}[z_{k}-H_{k}{\overset{⌢}{x}}_{k}^{-}]$$

(41) $$P_{k}^{+}=[I-K_{k}H_{k}]P_{k}^{-}$$

The error states are described, as shown in Equation (42)

(42) $$\delta x^{\prime }=[\begin{array}{ccccccc} \delta P_{1\times 3} & \delta v_{1\times 3} & \delta \theta_{1\times 3} & b_{a1\times 3} & b_{g1\times 3} & S_{a1\times 3} & S_{g1\times 3} \end{array}]$$

where the first three elements of the vector are the navigation error states which are 3D position, 3D velocity, and three attitude angles (roll, pitch, and azimuth) respectively; the other four elements are the biases and scale factors of the accelerometers and gyroscopes. *b_a_* and *b_g_* are the bias drift of the accelerometers and gyroscopes in *x*, *y*, and *z* directions respectively, and *S_a_* and *S_g_* are the scale factor of the 3D accelerometers and the 3D gyroscopes respectively.

### 5.2. Navigation System Integration Architecture

The proposed navigation system integrates onboard navigation sensors (GNSS/IMU) with CPD sensors (triaxial accelerometers) and vehicle forward velocity from OBD-II. In the proposed navigation system, GNSS-derived positions and velocities are integrated with IMU through EKF. The IMU is also used to provide navigation information during GNSS signal outages, and can be used for fast GNSS signal reacquisition. In this case, and to control IMU drift, the heading change estimated from the CPD accelerometers and the vehicle forward velocity provided through OBD-II are used as update measurements in EKF.

**Figure 8:**
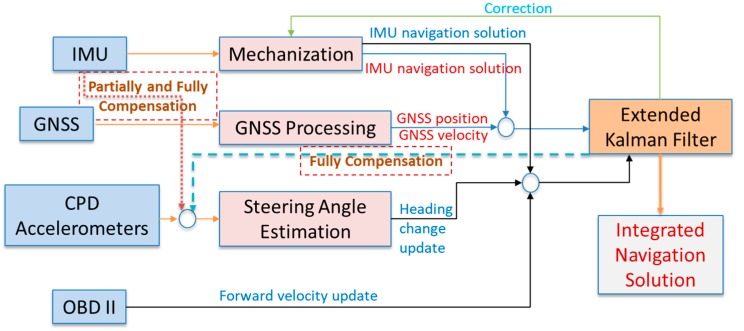
Navigation system with different compensation for the steering angle estimation method.

For the partially-compensated steering angle estimation method, additional static leveling compensation is applied to correct the inclination angle of the steering wheel before computing the steering angle. For the fully-compensated steering angle determination method, the onboard accelerometer, as well as the pitch angle that is estimated using EKF, will contribute to making steering angle estimations. Finally, the non-compensated steering angle estimation method does not take into account any of the previous mentioned factors. Figure 8 shows the proposed navigation scheme with different compensation level of the steering angle estimation method.

## 6. Experimental Results

A land vehicle test was conducted at the city of Calgary. The onboard navigation system includes the Pixhawk (Px4), which consists of Invensense MPU-6000 and a u-blox GPS single frequency receiver (LEA-6H module), while the CPD was the iPad air, which was mounted onto the driving steering wheel of a Ford Focus car. An OBD-II interface (Uni-link Mini ELM327 OBD-II Bluetooth Scanner Tool) was used in the experiment to access information about the vehicle’s forward velocity.

The data rate of the Pixhawk IMU is 25 Hz, while that of the GPS is 5 Hz. On the other hand, the iPad IMU’s data rate is 50 Hz, which is acquired by SensorLog software application, and that of the OBD-II is 1 Hz. In our tests, the change of heading updates estimated from the iPad were downsampled to 5 Hz during navigation solution estimations in the EKF.

### 6.1. Steering Angle and Heading Change Results Using CPD Accelerometers

**Figure 9:**
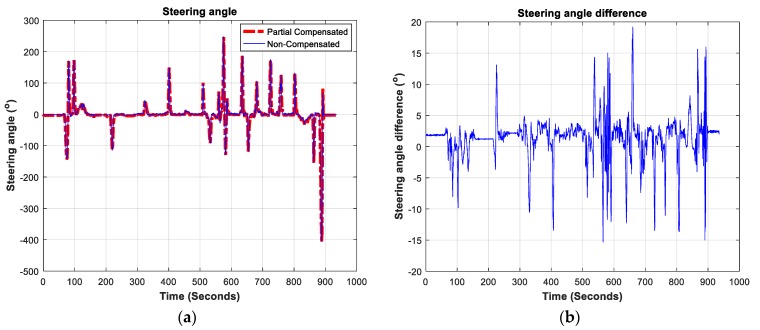
(**a**) Partially- and non-compensated steering angle estimation method; (**b**) the steering angle difference between the partially and non-compensated steering angle estimation methods.

As described, steering angle and heading changes can be estimated by three methods: non-compensated, partially-compensated, and fully-compensated. The non-compensated method doesn’t consider any of the previously mentioned factors that may render steering angle estimations less effective. The partially-compensated method considers the static leveling of the onboard IMU accelerometers, as well as the inclination angle of the steering wheel and the acceleration of the vehicle; it doesn’t consider dynamic changes in the vehicle’s inclination. Finally, the fully-compensated method takes into consideration all the aforementioned factors. These three methods were compared in this test. Figure 9 shows steering angle determinations using the partially and non-compensated methods and the difference between them. The figure clearly show that the two methods generally offer similar trends.

The maximum difference between the non- and partially-compensated steering angle estimates is around 19°. The Root Mean Square (RMS) of the difference between these two methods is 3.6436° for the steering angle difference and 0.2277° for the change of heading difference.

Table 1 lists the RMS of the steering angle and change of heading differences between the non-, partially- and fully-compensated methods.

As shown in Table 1, there is a significant difference between the non- and the partially-compensated methods’ estimates: the difference is slightly higher between the non- and the fully-compensated methods. However, there is a slight difference between the partially- and the fully-compensated methods.

The difference between the change of heading estimated by the three methods and the reference heading change determined by the GNSS/INS integration were computed to evaluate the proposed techniques. Table 2 shows the Root Mean Square Error (RMSE) of the three proposed methods.

**Figure 10:**
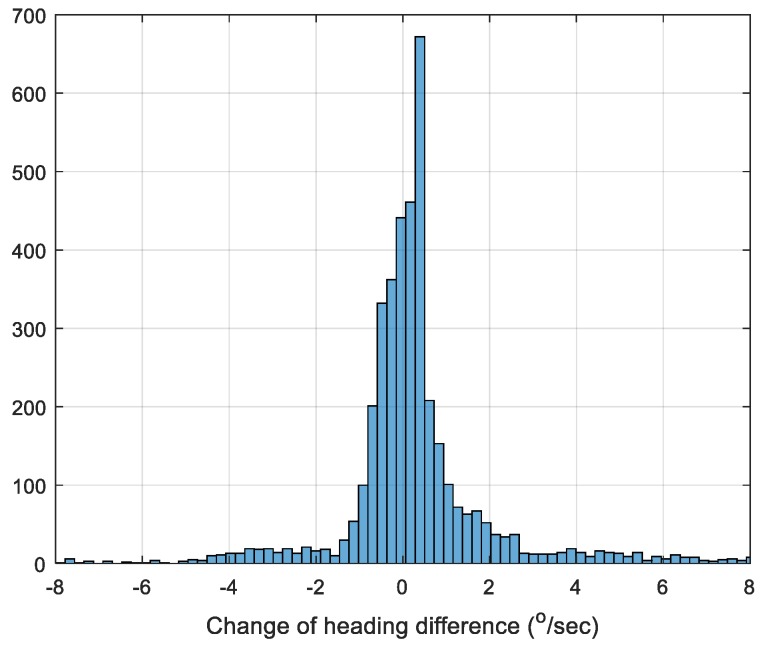
Histogram of the heading change difference between the fully-compensated method and GNSS/INS integrated solution.

Figure 10 depicts the histogram of the change of heading difference between the fully-compensated method and the GNSS/INS integrated solution.

The three methods are used in navigation state estimations using EKF through changes of heading updates to show the effect of each method on the final estimated navigation states, as will be discussed in the next section.

### 6.2. Navigation States Estimation Using EKF

This section is divided into two subsections. The first shows the effect of each steering angle estimation method on the final integrated navigation solution, while the second shows the effect of different updates on the final estimated navigation states.

#### 6.2.1. Effect of the Three Steering Angle Estimation Methods on the Navigation Solution

Changes of heading are computed through the three methods to show the effect of each method on the final navigation solution. The change of heading contributes to integrated navigation estimations as a relative heading update where the heading at time (*k* + 1) is equal to the heading at time (*k*) plus the change of heading (Δ*θ_vehicle_*) as given in Equation (43).

(43) $$Heading_{k+1}=Heading_{k}+\Delta \theta_{vehicle}$$

The vehicle steering ratio, in our case for the Ford Focus, is (1:16); this was provided in the manufacture’s specifications.

**Figure 11:**
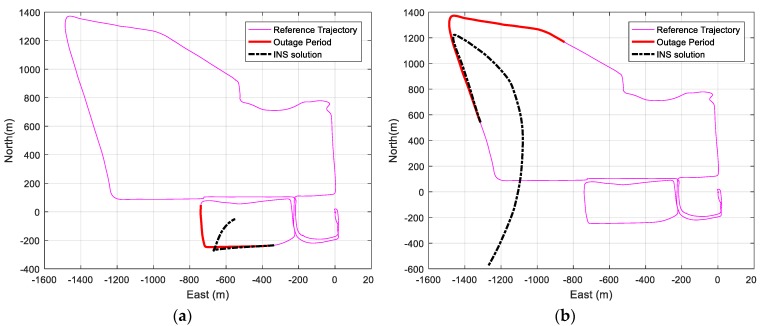
(**a**) Trajectory IMU standalone navigation solution for 60 s GNSS signal outage for a sample outage region. (**b**) Trajectory IMU standalone navigation solution for 120 s GNSS signal outage for another sample outage region.

First, the navigation solution is estimated using the Pixhawk (Px4) GNSS/IMU integrated system as the reference navigation, which has an expected sub-meter level accuracy. Then, different simulated GNSS signal outages are applied for 60 s and 120 s. During these periods, the IMU works in stand-alone mode to provide the navigation solution. Figure 11 shows the reference trajectory, the GNSS signal outage region, and the IMU stand-alone navigation solution for the periods of 60 and 120 s GNSS signal outage for two different sample outage regions.

As shown in Figure 11, the IMU navigation solution deviates considerably from the reference navigation solution due to the large bias drifts of the accelerometers and gyroscopes. The average RMSE of the position of the IMU stand-alone navigation solution for different GNSS signal outages is 98.97 m for 60 s GNSS signal outage and 392.105 m for 120 s GNSS signal blockage.

**Figure 12:**
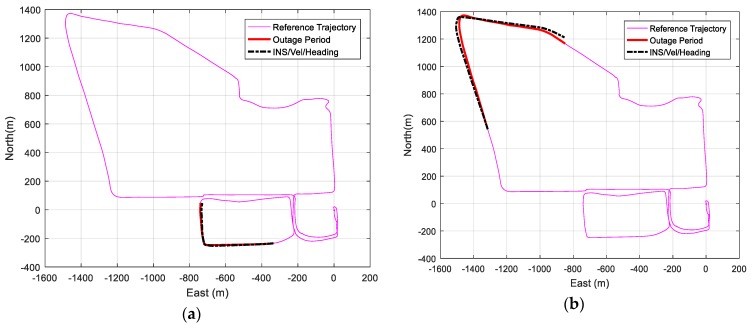
(**a**) Trajectory IMU/ fully-compensated heading change update/velocity update integrated navigation solution for 60 s GNSS signal outage for a sample outage region. (**b**) Trajectory IMU/ fully-compensated heading change update/velocity update integrated navigation solution for 120 s GNSS signal outage for a sample outage region.

Change of heading and velocity updates are then applied to aid the IMU during GNSS signal outages. The three developed methods of steering angle and change of heading estimation are used separately in the navigation solution. Figure 12 shows the reference trajectory with the outage period and the IMU/vehicle forward velocity/change of heading integrated navigation solution for 60 and 120 s GNSS signal blockage when using the fully-compensated steering angle estimation method for different outage regions.

Figure 12 shows that the integrated navigation solution is very close to the reference solution and provides more accurate solutions compared to the IMU standalone navigation during GNSS signal outages.

Two other integrated navigation solutions were tested using the other two steering angle estimation methods, i.e., the non- and partially-compensated methods, to compare the final integrated navigation position states estimations. Table 3 lists the average RMSE of the three methods for different outage periods.

The fully-compensated steering angle estimation method provides the best navigation results, reaching 5.47 m for 60 s outage and an average RMSE of 14.18 m for 120 s GNSS signal outage. However, there is a slight difference between the partially- and fully-compensated methods. Such a difference could be attributed to the fact that the test area does not exhibit a high level of variation of height. The non-compensated method provides the least accurate position solution. However, the performance variation between the methods is expected to increase under more extreme acceleration patterns and high height variations in the vehicle’s motion.

A comparison between the results with different factors being taken into account was included to assess whether the extra hardware/processing required to consider all factors is justified and can significantly affect the final results. The comparison results indicate that the sole use of CPD (without any usage of external information from the onboard IMU) could achieve comparable results to that of a fully integrated system.

#### 6.2.2. Effect of Different Updates on the Navigation Solution

Different updates can contribute to integrated navigation estimations during GNSS signal outages by limiting the IMU drift. These updates include velocity updates from OBD-II and heading change updates from steering angle calculations. Different combinations of updates for navigation estimations were tested in order to study the effect of each update on the final navigation solution.

Table 4 describes the average RMSE for different GNSS signal outage regions of the integrated navigation solution using the IMU standalone, IMU/velocity update integration, and IMU/forward velocity updates/proposed change of heading updates navigation solutions.

Table 4 shows that integrating the IMU standalone solution with the velocity and heading change updates provides more accurate navigation solutions than the IMU/forward velocity integrated solution. The position RMSE of the proposed heading update, along with the velocity update when aiding IMU, reaches 8.56 m and 15.33 m for 60 and 120 s GNSS signal outage respectively. On the other hand, the RMSE of the IMU /forward velocity integrated solution reaches 9.20 m and 16.10 m for 60 s and 120 s GNSS signal outage respectively.

It was observed that the change of heading updates slightly enhance the solution when they are provided to the navigation filter. However, in a GNSS denied environment, land vehicle navigation solution accuracy depends on the accuracy of the INS, the availability and the accuracy of velocity data, and heading change updates. The INS accuracy is based on the precision of the INS parameters during the GNSS availability period. However, if such a condition is not fulfilled, the quality of the navigation solution will deteriorate, and the heading change role will be able more significantly improve the solution. Moreover, an accurate change of heading estimation can help in achieving a comparable solution without an extra INS system i.e., using the proposed heading change estimation method with the vehicle velocity being obtained from a commercial OBD II to form a dead reckoning system.

## 7. Conclusions

Consumer portable device accelerometers are employed to improve the accuracy of a low-cost vehicle onboard IMU during GNSS signal outages by estimating heading changes of the vehicle, which serve as updates through the EKF to mitigate the IMU drift. Changes of heading are estimated by calculating the steering wheel angle through three methods: non-compensated, partially-compensated, and fully-compensated steering angle estimation. There are four main factors that influence the steering angle computation: the static leveling of the onboard IMU in vehicle stationary mode, the inclination angle of the vehicle’s steering wheel, the vehicle acceleration, and the changes of vehicle inclination due to vehicle motion on up- or down-hill roads, which affects the vehicle’s acceleration, as measured across the vehicle longitudinal axis. An experimental test was conducted, and the navigation solution was improved to obtain an accuracy 91% for 60 seconds of GNSS signal outage when providing the navigation filter with the proposed change of heading and velocity updates when compared with the IMU standalone solution.

The proposed change of heading estimation methods are based on the use of CPD accelerometers only. Other sensors such as gyroscopes and/or magnetometers are not used to avoid gyro drift and magnetometer interference problems. Moreover, the estimation method doesn’t require any external dedicated hardware/software to acquire the steering wheel angle, which reduces the system cost. Finally, the proposed steering wheel angle estimation is not used only for land vehicle navigation enhancement, but may also be employed in driving safety applications.

## Figures and Tables

**Table 1:** RMS of the different steering angle and change of heading estimation methods.

Steering Angle Estimation Method	RMS (°)	
	Steering Angle	Change of Heading
Non-Compensated and Partially-Compensated	3.6436	0.2277
Non-Compensated and Fully-Compensated	3.6469	0.2279
Partially-Compensated and Fully-Compensated	0.0221	0.0014

**Table 2:** RMSE of the different change of heading estimation methods.

Heading Change Estimation Method	RMSE (°/s)
Non-Compensated	1.77
Partially-compensated	1.71
Fully-compensated	1.70

**Table 3:** Average RMSE of the integrated 2D position states for heading change and velocity updates for different GNSS signal outage periods.

Steering Angle Estimation Method	RMSE (m)	
	60 s Outage	120 s Outage
Non-Compensated	5.58	14.68
Partially-Compensated	5.48	14.19
Fully-Compensated	5.47	14.18

**Table 4:** Average RMSE for different GNSS signal outage regions of the integrated 2D position states for different types of updates.

Type of Solution	RMSE (m)	
	60 s Outage	120 s Outage
IMU standalone	97.98	493.49
IMU/velocity update	9.20	16.10
IMU/velocity/heading change updates	8.56	15.32
